# Evaluation of two study demands-resources-based interventions: a randomized controlled trial

**DOI:** 10.3389/fpsyg.2024.1368267

**Published:** 2024-06-10

**Authors:** Lorena Sarah Körner, Timo Kortsch, Kerstin Rieder, Thomas Rigotti

**Affiliations:** ^1^Department of Business Psychology, Aalen University of Applied Sciences, Aalen, Germany; ^2^Department of Social Sciences, IU International University of Applied Sciences, Erfurt, Germany; ^3^Department of Work, Organizational, and Business Psychology, Institute for Psychology, Johannes Gutenberg University Mainz, Mainz, Germany; ^4^Leibniz Institute for Resilience Research, Mainz, Germany

**Keywords:** study demands-resources framework, online intervention, mindfulness, study crafting, self-undermining, exhaustion, randomized controlled trial

## Abstract

**Introduction:**

Higher education students experience significant levels of exhaustion in their studies, yet there are limited evidence-based support programs available. Therefore, this study evaluated a novel intervention approach by testing the effectiveness of two online interventions based on the study demands-resources framework. These interventions aimed to balance demands and resources. Derived from the theoretical assumptions of the framework, we hypothesized that the interventions would increase study and personal resources, engagement, and study crafting, and decrease study demands, exhaustion, and self-undermining. Additionally, we hypothesized that demands and resources would mediate the effects of the intervention on engagement, exhaustion, study crafting, and self-undermining.

**Methods:**

Conducted as a randomized controlled trial with a waitlist control group (*n* = 71), the study involved participants in two intervention groups who engaged with the interventions for 2 weeks. Intervention group 1 (*n* = 64) focused on adapting demands, while intervention group 2 (*n* = 70) focused on increasing resources. The design allowed for a comparison of the effectiveness of these different approaches. Participants completed questionnaires before and after the intervention, and at a 5-week follow-up.

**Results:**

Results of the analyses of variance with repeated measures revealed that the interventions had significant positive effects on the personal resource mindfulness, two study crafting strategies, self-undermining, and exhaustion. Notably, intervention group 2 exhibited more positive outcomes. The hypothesized mediation effects through mindfulness were partially supported.

**Discussion:**

The study demonstrates the considerable potential of interventions based on the study demands-resources framework for higher education institutions in supporting student well-being.

## 1 Introduction

In a representative survey among German higher education students, more than half reported experiencing high levels of stress (Herbst et al., 2016). Stress levels in students significantly rise throughout the semester, peaking in (pre-)exam weeks (Pitt et al., 2018). Moreover, an international review indicated that 55% of students suffer from emotional exhaustion, the primary symptom of burnout (Rosales-Ricardo et al., 2021). Meta-analytic studies indicate that burnout negatively impacts academic performance (Madigan and Curran, 2021). Burnout is further a negative predictor of dropout intention (Marôco et al., 2020). Beyond academic implications, stress and burnout can also have detrimental effects on physical health and may contribute to the development and persistence of mental disorders (Pascoe et al., 2020). The COVID-19 pandemic has further exacerbated stress levels of students by reducing their resources such as social interaction and support, and increasing their demands such as workload and self-study issues (Hoss et al., 2021; Tsiouris et al., 2023).

Thus, there is an urgent need to develop and evaluate interventions aimed at reducing stress and exhaustion among students, while enhancing their coping skills (e.g., Pascoe et al., 2020; Madigan and Curran, 2021). Interventions that focus on reducing demands and enhancing resources have been considered as particularly effective in this regard (Jagodics and Szabó, 2022). The study demands-resources (SD-R) framework, an adaption of the job demands-resources (JD-R) model from the work context, explains the interplay between demands and resources and their impact on student exhaustion and engagement, as well as their proactive (i.e., study crafting) and dysfunctional (i.e., self-undermining) behavior (Bakker and Demerouti, 2014; Lesener et al., 2020; Körner et al., 2021). The JD-R model has been successfully applied in the work context by serving as a theoretical basis for interventions designed to enhance employee well-being (Bakker and Demerouti, 2014, 2017). However, there is a gap in the application of the SD-R framework. Interventions based on this framework, aimed at balancing demands and resources to enhance student well-being are lacking.

The end of the semester, characterized by an accumulation of stressors, is an especially critical time to implement such interventions to prevent negative stress-related outcomes (Pitt et al., 2018). In a qualitative study, students further expressed interest in compact interventions, which can be more easily integrated into their daily study routines, particularly before high-stress periods like exams (Seidl et al., 2018). Furthermore, students prefer online interventions due to their anonymity, accessibility, and time and location flexibility (Lutz-Kopp et al., 2019).

Thus, the aim of this study is to evaluate the efficacy of two brief online SD-R-based interventions aimed at improving student well-being. We conducted a randomized controlled trial with a waitlist control group and a follow-up assessment during the pre-exam period. This was to evaluate the sustainability of intervention effects and determine if the interventions could prevent negative outcomes typically occurring during the exam phase. Our study thereby aims to make three key contributions: First, we evaluated the efficacy of two SD-R-based online interventions aimed at balancing demands and resources. We examined the impact of these interventions on study resources and demands, engagement, and exhaustion, as well as the underlying mediation processes. Second, we also investigated the impact of the SD-R-based interventions on personal resources, study crafting, and self-undermining. Since these variables have been relatively underexplored in research on the SD-R framework, our study expands the framework, drawing parallels to the JD-R model where these aspects have received more extensive research attention (e.g., Xanthopoulou et al., 2007; Tims et al., 2013; Bakker and Wang, 2020; Bakker et al., 2023a). Third, our study addresses the critical need for evidence-based student support programs, as highlighted by recent research (e.g., Mülder et al., 2022). By evaluating a novel theoretical approach—the SD-R framework—through brief online interventions, we provide new perspectives on effective strategies to improve student well-being. Additionally, incorporating a follow-up measurement enabled us to assess the sustainable impact of these interventions. Taken together, our research contributes to the practical validation of the SD-R framework by testing how SD-R-based interventions can actively influence the key processes specified in the framework.

## 2 Theoretical background

### 2.1 Demands and resources in the study demands-resources framework

The SD-R framework, derived from the well-established JD-R model in the work context (Demerouti et al., 2001; Bakker and Demerouti, 2014), includes two central paths. The health-impairment path posits that study demands are positively related to burnout, while the motivational path posits that study resources are positively related to engagement and negatively related to burnout (Lesener et al., 2020). The validity of these two paths is supported by broad empirical evidence from cross-sectional (e.g., Gusy et al., 2016), longitudinal (e.g., Gusy et al., 2021), and diary studies (e.g., Körner et al., 2021).

Study demands occur at an organizational, physical, social, or psychological level. They require high levels of physical or mental effort, and are therefore associated with physiological or psychological costs (Lesener et al., 2020). Overload and time pressure are among the most common study demands and key predictors of burnout within the SD-R framework (e.g., Lesener et al., 2020; Gusy et al., 2021). Consequently, our study examined the *psychological demands* of studying. This study demand encompasses aspects such as time pressure, haste, or competing tasks (Schmidt et al., 2019).

Study resources also occur at organizational, physical, social, or psychological levels and can help achieve goals, promote personal development, and reduce study demands (Lesener et al., 2020). An important study resource, and a strong predictor of engagement within the SD-R framework even during the COVID-19 pandemic, is *social support from lecturers* (Salmela-Aro et al., 2022). In line, Reichel et al. (2023) assume that social support from lecturers plays an especially important role in times of numerous stressors, such as during a pandemic. We therefore examined this study resource in our study. Social support from lecturers includes the extent to which lecturers/professors take an interest, help students in their studies and support them through good organization.

The JD-R model has been extended to incorporate personal resources alongside job resources (Bakker and Demerouti, 2017). Personal resources are self-aspects associated with resilience and a sense of being able to successfully control and influence the environment, even in difficult situations (Hobfoll et al., 2003). These resources help to achieve goals, grow personally, and protect against threats (Xanthopoulou et al., 2009). A key personal resource receiving increased attention in recent years is mindfulness, defined as the “enhanced attention to and awareness of current experience or present reality” (Brown and Ryan, 2003, p. 822). Grover et al. (2017) integrated mindfulness into the JD-R model, highlighting its relevance as a personal resource within this model. In the academic context, a positive relationship between mindfulness and engagement as well as a negative relationship between mindfulness and burnout was found (Robins et al., 2015), demonstrating its impact similar to that of study resources within the SD-R framework. Consequently, our study examined the personal resource of *mindfulness*.

According to the SD-R framework, engagement is fostered by high levels of study and personal resources (Ouweneel et al., 2011; Lesener et al., 2020). Engagement is a fulfilling, positive state that encompasses the three dimensions of vigor, dedication, and absorption. Vigor includes high levels of energy and perseverance and a willingness to try hard even when difficulties arise. Dedication includes feelings of enthusiasm, inspiration, and pride. Absorption is a state of concentration and flow (Schaufeli et al., 2002). In the academic context, engagement is of great importance due to its positive relationship with academic performance (Salanova et al., 2010) and life satisfaction (Lesener et al., 2020).

Conversely, the SD-R framework posits that burnout results from high study demands and a lack of study resources (Lesener et al., 2020). Burnout encompasses the three dimensions of exhaustion, cynicism, and professional inefficacy. Exhaustion describes a feeling of fatigue due to high study demands and represents the core dimension of burnout. Cynicism refers to a detached attitude toward one’s studies, and professional inefficacy refers to a feeling of incompetence as a student (Schaufeli et al., 2002).

### 2.2 Study crafting and self-undermining in the study demands-resources framework

The JD-R model has been further expanded to incorporate two behavioral variables: job crafting within the motivational path (Tims and Bakker, 2010) and self-undermining within the health-impairment path (Bakker and Wang, 2020). Recently, the concept of job crafting has been adapted to the academic context as *study crafting* and incorporated into the SD-R framework (Körner et al., 2021). Study crafting refers to the proactive adjustments students make to their study environment, aligning their studies with their personal skills and preferences (Körner et al., 2021). This concept draws from the job crafting strategies distinguished by Tims et al. (2013), and includes four analogous study crafting strategies: Increasing structural resources, increasing social resources, increasing challenging demands, and decreasing hindering demands. Increasing structural resources involves activities that contribute to personal growth, such as skill development. Increasing social resources includes behaviors such as seeking feedback or advice. Increasing challenging demands involves activities such as taking on additional projects or attending extra lectures. Decreasing hindering demands involves, for example, trying to make study less demanding (Tims et al., 2012; Körner et al., 2021).

On the other hand, the concept of self-undermining, defined as “behavior that creates obstacles that may undermine performance” (Bakker and Costa, 2014, p. 115), has been included in the JD-R model. Self-undermining involves behaviors such as making mistakes or provoking conflicts (Bakker and Wang, 2020). This concept has received little attention within the academic context so far. However, an initial study investigated self-undermining within the SD-R framework and confirmed that the health-impairment path can be extended to include this concept (Körner et al., 2021).

### 2.3 Gain cycles and loss cycles

Recent versions of the JD-R model specify a gain cycle within the motivational path and a loss cycle within the health-impairment path, further clarifying the interplay among JD-R variables (Bakker et al., 2023a). The model assumes that engaged employees want to maintain their engagement and therefore attempt to build new resources through job crafting. The resources built through job crafting, in turn, foster engagement, resulting in a gain cycle with reciprocal relationships between resources, engagement, and job crafting (Bakker et al., 2023a). Empirical evidence supports this assumption. Systematic reviews and meta-analyses in the work context have affirmed positive relationships between job crafting and various job resources (e.g., autonomy, social support), between job crafting and engagement, as well as between job resources and engagement (Rudolph et al., 2017; Lesener et al., 2019; Lichtenthaler and Fischbach, 2019; Zhang and Parker, 2019). In the academic context, similar positive correlations have been observed between study and personal resources and engagement (Ouweneel et al., 2011; Lesener et al., 2020). Furthermore, a weekly diary study found a positive relationship between study resources and study crafting, mediated by engagement (Körner et al., 2021).

Conversely, the JD-R model suggests that exhausted employees tend to engage in self-undermining. Through self-undermining, they create new demands and obstacles, which further increase exhaustion, resulting in a loss cycle (Bakker et al., 2023a). This assumption is empirically supported by research indicating a positive reciprocal relationship between job demands and burnout and a positive relationship between exhaustion and self-undermining as well as between job demands and self-undermining (Lesener et al., 2019; Rațiu and Dobre, 2020; Bakker et al., 2023b). Within the academic context, the concept of self-undermining is relatively underexplored. However, preliminary research indicated a positive relationship between study demands and self-undermining via exhaustion at the weekly within-person level (Körner et al., 2021).

### 2.4 Interventions based on the job demands-resources model and the study demands-resources framework

In the work context, the JD-R model has been effectively utilized as a theoretical foundation for interventions aimed at enhancing employee well-being (Bakker and Demerouti, 2017; Bakker et al., 2023a). This model provides various starting points for such interventions, including optimizing job demands, increasing job and personal resources, and promoting job crafting (Bakker and Demerouti, 2014; Bakker et al., 2023a). Research has validated the effectiveness of these interventions, demonstrating significant increases in personal resources, engagement, and job crafting, as well as reductions in exhaustion (Bakker, 2017; van Wingerden et al., 2017; Gordon et al., 2018; Bakker and van Wingerden, 2021). To our knowledge, only one intervention has employed the SD-R framework as a theoretical foundation for an intervention within the academic context so far. This intervention, a study crafting intervention, was adapted from job crafting interventions in the work context. It successfully increased study crafting while concurrently fostering engagement and reducing exhaustion (Körner et al., 2022).

Our present study expands this research by again utilizing the SD-R framework for developing interventions, but with different starting points within the SD-R framework compared to the previous study crafting intervention. Whereas the study crafting intervention primarily focused on enhancing study crafting, our current interventions aim to balance demands and resources. Besides the impact on demands and resources, we also investigate the influence of the interventions on the other variables of the SD-R framework. Drawing from the theoretical assumptions and empirical findings from both the JD-R model and the SD-R framework, and building on results from job crafting and study crafting interventions, we hypothesize:

*Hypothesis 1 (H1)*: In the intervention groups (IGs), there will be a significant increase in the levels of (a) the study resource social support from lecturers and (b) the personal resource mindfulness, and a significant decrease in the level of (c) the study demand psychological demands after the intervention (T2 and T3), compared to the levels before the intervention (T1) and compared to the waitlist control group (WLC).

*Hypothesis 2 (H2)*: In the IGs, there will be a significant increase in the level of (a) engagement, and a significant decrease in the level of (b) exhaustion after the intervention (T2 and T3), compared to the levels before the intervention (T1) and compared to the WLC.

*Hypothesis 3 (H3)*: In the IGs, there will be a significant increase in the level of (a) study crafting, and a significant decrease in the level of (b) self-undermining after the intervention (T2 and T3), compared to the levels before the intervention (T1) and compared to the WLC.

We also explore the underlying mechanisms that influence the outcomes of our intervention. Research into these mediating processes of interventions based on the JD-R model or the SD-R framework remains relatively scarce. However, in the work context, there is evidence suggesting that job crafting serves as a mediator in the relationship between job crafting interventions and JD-R outcomes like engagement (Mukherjee and Dhar, 2023). Similarly, an initial study in the academic context has found comparable results, with study crafting acting as a mediator in the effects of a study crafting intervention on engagement and exhaustion (Körner et al., 2022). Our SD-R-based interventions are targeted at balancing demands and resources. Therefore, we assume that the interventions will primarily influence these variables, which in turn, will affect other variables within the SD-R framework. Thus, we hypothesize:

*Hypothesis 4 (H4)*: Study and personal resources will mediate the relationship between the intervention and (a) engagement, (b) exhaustion, (c) study crafting, and (d) self-undermining.

*Hypothesis 5 (H5)*: Study demands will mediate the relationship between the intervention and (a) engagement, (b) exhaustion, (c) study crafting, and (d) self-undermining.

## 3 Methods

### 3.1 Participants and procedure

The study was conducted at Aalen University. All students received an email invitation to participate in the study, we presented the study in selected lectures, promoted it at a trade fair, and through social media. Inclusion criteria were a minimum age of 18 years and enrollment at Aalen University. The exclusion criterion was a diagnosed mental disorder. After registration, students received detailed participant information. The study was conducted in accordance with the ethical guidelines of the American Psychological Association: Participation was voluntary and could be terminated at any time without giving a reason. There was no monetary compensation, but students received credit for their participation and got access to all modules of the online intervention upon completion of the study. Informed consent was obtained from all participants before the study began.

**Figure 1:**
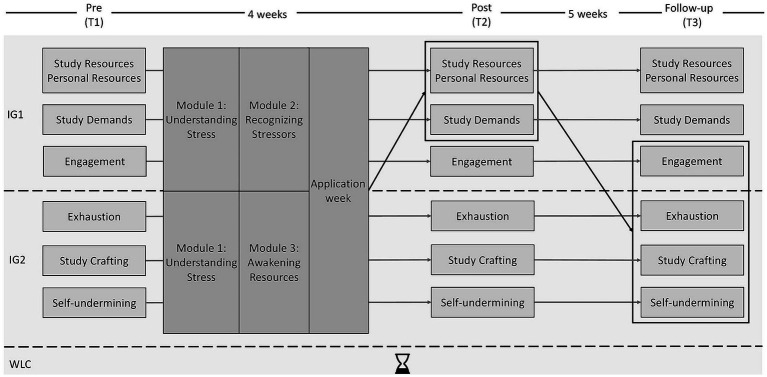
Study design and hypotheses. Gray arrows represent the hypothesized changes in the variables over the measurement time points. For study resources (social support from lecturers), personal resources (mindfulness), engagement, and study crafting (increasing structural resources, increasing social resources, increasing challenging demands, decreasing hindering demands), an increase was hypothesized. For study demands (psychological demands), exhaustion, and self-undermining, a decrease was hypothesized. Black arrows represent the hypothesized mediation effects.

The study was a randomized controlled trial with two IGs and one WLC. Students were randomly assigned to one of the three groups after enrollment. In the week prior to the start of the intervention, students received information about their group assignment, access information to the online platform, and the link to the T1 questionnaire by e-mail. In the first intervention week, the Introductory Module and Module 1 (“Understanding Stress”) were unlocked for both IGs. In the second intervention week, IG1 received access to Module 2 (“Recognizing Stressors”) and IG2 received access to Module 3 (“Awakening Resources”). During the third week, students were asked to apply what they had learned in the two modules to their daily routines. Students then completed the T2 questionnaire and 5 weeks later the T3 questionnaire. It is important to note that due to the COVID-19 pandemic and the increasing number of infections, most of the lectures changed from face-to-face to online from T1 to T2. The T3 questionnaire was completed 2 weeks prior to the start of the exam period and lectures continued to be delivered online. Figure 1 shows the study design.

We conducted an *a priori* power analysis for a repeated measures analysis of variance in a 3×3 factorial design to estimate the required sample size. We assumed a small effect size of *f* = 0.1, based on findings of previous job crafting interventions, and targeted a power of 0.80. This resulted in a sample size of 204. A total of 253 students enrolled in the study of whom 242 completed the T1 questionnaire (95.7% response rate), 220 completed the T2 questionnaire (9.1% dropout from T1 to T2), and 208 completed the T3 questionnaire (5.5% dropout from T2 to T3). The overall dropout rate was 18.7%, which is relatively low compared to other online interventions, which report adherence rates of about 50% (Kelders et al., 2012) and attrition rates of 40–50% (Bennett and Glasgow, 2009; Kuster et al., 2017). Previous studies have pinpointed various factors contributing to dropout in online interventions, such as dwindling interest over time (Bennett and Glasgow, 2009), the burden of high time demands, and uncertainties related to the intervention’s content and instructions (Fasthoff et al., 2023). Given that our intervention took place just before the Christmas period and exam season, it is plausible to suggest that the observed dropout could especially be linked to heightened demands and the resultant time constraints.

**Figure 2:**
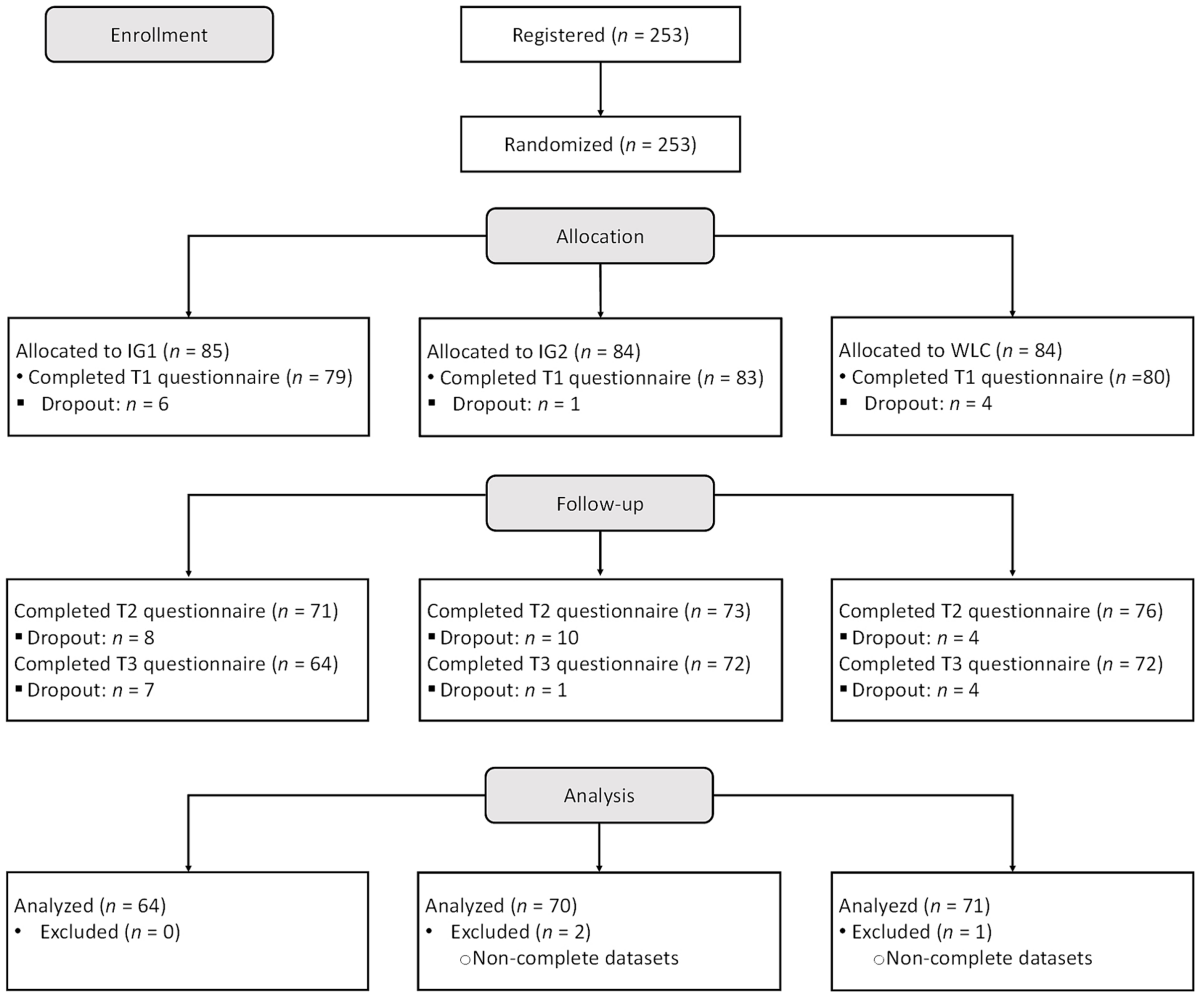
COSORT flowchart of participants.

Our final sample consisted of 205 students, distributed across the two IGs and the WLC, who completed the three questionnaires. Of these, 141 were female, 62 were male, 1 was diverse, and 1 did not provide gender information. The participants studied in five faculties (chemistry: *n* = 12, electronics and informatics: *n* = 12, mechanical and materials engineering: *n* = 19, optics and mechatronics: *n* = 30, economics: *n* = 132). They were on average 21.82 years old (*SD* = 3.08) and the majority of participants (*n* = 199) were in a bachelor’s degree program (master’s degree program: *n* = 6). On average, the students were in their third study semester (*M* = 3.25, *SD* = 2.61). IG1 consisted of 64 students. Of these, 44 were female and 20 were male with a mean age of 21.94 years (*SD* = 3.57). IG2 consisted of 70 students. Of these, 47 were female and 21 male (diverse: *n* = 1, not specified: *n* = 1) with a mean age of 21.50 years (*SD* = 2.60). The WLC consisted of 71 students. Of these, 50 were female and 21 were male with a mean age of 22.04 years (*SD* = 3.07). Figure 2 shows the study procedure.

### 3.2 The interventions

Our study utilized two interventions based on the “Einfach weniger Stress [Simply less stress]” (EWS) concept (Paulsen and Kortsch, 2020), which has been tested and certified by the Zentrale Prüfstelle Prävention [Central Examination Office for Prevention] (ZPP; www.zentrale-pruefstelle-praevention.de).^1^ The concept has already been successfully applied in the work context (Fasthoff et al., 2023). Initially designed as a face-to-face training, EWS was adapted into an online format to enhance accessibility and increase its reach. The web-based version of the intervention was implemented as a standalone Wordpress-based solution. The EWS intervention includes five modules: (1) understanding Stress, (2) recognizing stressors, (3) awakening resources, (4) planning implementation, (5) acting calmly. The modules build on each other but are carefully crafted to be self-sufficient, offering a complete package of content. Every module starts with clearly defined learning objectives and concludes with a comprehensive review, coupled with an observation task designed to integrate the learned content into daily life. In each module, exercises address at least one Behavior Change Technique, as outlined by Michie et al. (2015) from one of four main categories: goals and planning (main category 1), observing behavior and giving feedback (main category 2), building knowledge (main category 4) or repeating and generalizing (main category 8). This thoughtful design ensures that even standalone modules can deliver significant intervention effects.

To address students’ preference for concise interventions that seamlessly fit into their academic routines, we have deliberately chosen to limit the selection to two modules per group, as supported by the findings of Seidl et al. (2018). Our selection prioritized modules 1–3, which are fundamentally based on the SD-R framework. This choice was made to ensure that our interventions are in line with the aims of SD-R-based strategies, following the guidance provided by research on JD-R-based interventions (Bakker et al., 2014, 2023a).

Module 1 provides an in-depth explanation of the SD-R framework, highlighting the interplay between demands and resources. Module 2 encourages participants to identify and reflect on their demands, while Module 3 focuses on recognizing, enhancing, and activating their resources. Both IGs completed Module 1, providing all participants with foundational knowledge about the SD-R framework. IG1 then primarily focused on adapting demands, while IG2 then emphasized increasing resources. This design enabled us to compare the effectiveness of these two intervention approaches.

Each module consisted of theoretical input, exercises, reflection activities, and transfer tasks. Participants could download and print worksheets or complete them digitally. Two fictional characters guided participants through the online course. An additional third fictional student character was created especially for this study. Participants were allocated 1 week to complete each module, with the option to stop at any time and continue later. The completion time of module 1 was approximately 90 min, and that of module 2 and 3 were approximately 110 min each. Table 1 provides an overview of the intervention modules.

**Table 1:** The three EWS intervention modules.

Module	Goals	Theory	Practice
Understanding stress	Understanding stress and stress reactions. Understanding the transactional stress model. Reflecting on one’s own stress experiences and recognizing the consequences of stress. Understanding the interplay of demands and resources. Recognizing the personal boundary between study and private life.	Stress (eustress/distress), stress reactions (Selye, 1956). Transactional stress model (Lazarus and Folkman, 1984, 1987). Coping strategies (Lazarus, 1991). JD-R model (Demerouti et al., 2001).	Reflection on the stress level of the last days. Reflection on different stress situations and one’s own reaction to them. Identification of demands and resources based on the JD-R model. Reflection on constant accessibility.
Recognizing stressors	Understanding stressors and their classification. Understanding inner drivers and their role in the development of stress. Developing more advantageous thoughts for selected stressors.	Definition of stressors. Typical stressors. Personality traits (inner drivers) as potential stress amplifiers.	Reflection of a stress situation of the last days. Stressor radar to identify own stressors. Classification of own stressors (frequency/importance). Identification of own inner drivers. Re-interpretation of stress situations/inner drivers.
Awakening resources	Understanding resources and their classification. Identifying available resources and how to activate them. Identifying previously unconscious resources. Creating an inner strength picture.	Definition of resources. Typical resources. Resource activation techniques.	Reflection of a stress situation of the last days. Dream journey to enable metaphorical access to one’s own resources. Resource radar to identify own resources. Inner strength picture. Identification of techniques to activate resources.

### 3.3 Measures

The questionnaires were identical at the three measurement time points. In the T1 questionnaire, we also collected sociodemographic data.

*Social support from lecturers* was measured with five items (e.g., “My lecturers/professors support me through good organization.”) and *psychological demands* were measured with seven items (e.g., “My studies are hectic.”) of the questionnaire on Structural Study Conditions (Schmidt et al., 2019). Items were rated on a 4-point Likert-type scale (1 = *does not apply*, 4 = *does apply*).

*Mindfulness* was measured using the short version of the Freiburg Mindfulness Questionnaire (Walach et al., 2004). The questionnaire contains 14 items (e.g., “I am in touch with my experiences, here and now.”), which were rated on a 4-point Likert-type scale (1 = *almost never*, 4 = *almost always*).

*Study crafting* was measured with a shortened version of the German Job Crafting Scale (Lichtenthaler and Fischbach, 2016) adapted to the academic context. The sub dimensions increasing structural resources (e.g., “I try to develop my capabilities.”), increasing social resources (e.g., “I ask others for feedback on my performance in my studies.”), increasing challenging demands (e.g., “When there is not much to do in my studies, I see it as a chance to start new projects.”), and decreasing hindering demands (e.g., “I make sure that my studies are mentally less intense.”) were measured with four items each. Items were rated on a 5-point Likert-type scale (1 = *not at all true*, 5 = *completely true*).

*Self-undermining* was measured using the self-undermining scale (Bakker and Wang, 2020). We adapted the six items (e.g., “I make mistakes.”) to the academic context and participants rated them on a 7-point Likert-type scale (1 = *never*, 7 = *always*).

*Engagement* was measured using the Utrecht Work Engagement Scale – Student Form, which includes three items each for vigor, dedication, and absorption (Schaufeli et al., 2002). Items (e.g., “I am immersed in my studies.”) were rated on a 7-point Likert-type scale (1 = *never*, 7 = *always*).

*Exhaustion* was measured using the short German Maslach Burnout Inventory – Student Survey (Wörfel et al., 2015). The scale consists of three items (e.g., “I feel drained by my studies.”) which were rated on a 7-point Likert-type scale (1 = *never*, 7 = *always*).

### 3.4 Strategy of analysis

Data were analyzed using IBM SPSS 28 and R 4.1.0 with package welchADF (Villacorta, 2017). First, we used chi-square tests and *t*-tests to test for sociodemographic differences among the three groups and multivariate analysis of variance (ANOVA) to test for significant T1 differences in all research variables. We tested hypotheses 1–4 with 3×3 repeated measures (RM) ANOVA. Measurement time (T1, T2, and T3) was the within-subject factor and group (IG1, IG2, and WLC) was the between-subject factor. The requirements for RM ANOVA are homogeneity of error variances (tested with Levene’s test) and sphericity (tested with Mauchly’s test). If the sphericity assumption was violated, the Greenhouse–Geisser correction (for *ε* < 0.75) or the Huynh-Feldt correction (for *ε* > 0.75) was used. If there was a significant time x group interaction effect, the time effect was examined separately for each group. If there was a significant time effect, the differences between each of the two measurement time points (T1–T2, T1–T3, and T2–T3) were further examined. If there was no significant time × group interaction effect, the main time effect (also separately for the three groups) was examined. Hypotheses 5 and 6 were tested using Model 4 of the Hayes Process macro for SPSS (Hayes, 2017). Group membership was included as the independent variable. Demands and resources at T2 were each included as the mediator. Engagement, exhaustion, study crafting, or self-undermining at T3 were each included as the dependent variable. T1 scores of the mediator and dependent variable were included as control variables. Bootstrapping with 5,000 samples was used to calculate 95% confidence intervals.

## 4 Results

Testing for T1 differences in the study variables between the three groups revealed no significant group effect, *F*(24, 384) = 0.76, *p* = 0.78. Similarly, there were no significant differences between the three groups on the sociodemographic variables of gender, *χ*^2^(6) = 3.95, *p* = 0.68, age, *F*(2) = 0.61, *p* = 0.55, study degree, *χ*^2^(2) = 0.98, *p* = 0.61, work experience, *χ*^2^(2) = 44, *p* = 0.80, and study semester, *F*(2) = 1.30, *p* = 0.29. Table 2 shows the means, standard deviations, reliabilities, and correlations between the study variables at T1, T2, and T3.

**Table 2:** Means, standard deviations, correlations, and reliabilities for all study variables at the three measurement time points.

Variable	*M*	*SD*	1	2	3	4	5	6	7	8	9	10	11	12	13	14
1. Social support T1	3.17	0.48	(0.80)													
2. Psychological demands T1	2.74	0.51	0.04	(0.81)												
3. Mindfulness T1	2.61	0.43	0.08	−0.03	(0.81)											
4. Incr. structural resources T1	3.92	0.50	0.19^**^	0.11	0.36^**^	(0.69)										
5. Incr. social resources T1	2.59	0.72	0.10	0.06	0.16^*^	0.16^*^	(0.64)									
6. Incr. challenging demands T1	2.92	0.77	0.01	0.19^**^	0.20^**^	0.33^**^	0.32^**^	(0.61)								
7. Decr. hindering demands T1	2.75	0.83	0.07	0.02	0.05	−0.03	0.16^*^	0.13	(0.69)							
8. Self-undermining T1	3.10	0.81	−0.20^**^	0.24^**^	−0.21^**^	−0.13	−0.03	0.03	−0.13	(0.74)						
9. Engagement T1	4.37	1.00	0.34^**^	0.02	0.25^**^	0.41^**^	0.35^**^	0.31^**^	0.04	−0.19^**^	(0.91)					
10. Exhaustion T1	3.12	1.05	−0.15^*^	0.34^**^	−0.22^**^	−0.13	−0.24^**^	−0.11	−0.02	0.27^**^	−0.43^**^	(0.80)				
11. Social support T2	3.08	0.50	0.72^**^	−0.10	0.16^*^	0.22^**^	0.12	0.03	0.02	−0.23^**^	0.29^**^	−0.16^*^	(0.83)			
12. Psychological demands T2	2.81	0.46	−0.05	0.71^**^	−0.03	0.13	0.08	0.19^**^	0.02	0.21^**^	0.07	0.26^**^	−0.09	(0.78)		
13. Mindfulness T2	2.68	0.43	0.17^*^	0.08	0.71^**^	0.34^**^	0.22^**^	0.26^**^	0.19^**^	0.17^*^	0.30^**^	−0.17^*^	0.26^**^	−0.02	(0.85)	
14. Incr. structural resources T2	3.87	0.51	0.31^**^	0.07	0.23^**^	0.53^**^	0.17^*^	0.27^**^	0.01	−0.09	0.44^**^	−0.19^**^	0.32^**^	0.03	0.40^**^	(0.65)
15. Incr. social resources T2	2.61	0.75	0.03	0.10	0.08	0.03	0.67^**^	0.22^**^	0.11	0.11	0.25^**^	−0.10	0.05	0.08	0.20^**^	0.15^*^
16. Incr. challenging demands T2	2.94	0.76	0.02	0.14^*^	0.22^**^	0.27^**^	0.27^**^	0.65^**^	0.13	0.01	0.31^**^	−0.14^*^	0.09	0.12	0.38^**^	0.38^**^
17. Decr. hindering demands T2	2.95	0.77	0.04	0.07	0.11	0.12	0.10	0.22^**^	0.51^**^	−0.06	0.08	−0.06	−0.03	0.02	0.27^**^	0.20^**^
18. Self-undermining T2	3.19	0.81	−0.17^*^	0.20^**^	−0.12	−0.08	−0.04	0.02	−0.14^*^	0.69^**^	−0.22^**^	0.27^**^	−0.16^*^	0.24^**^	−0.20^**^	−0.17^*^
19. Engagement T2	4.34	1.06	0.34^**^	0.02	0.27^**^	0.39^**^	0.28^**^	0.24^**^	−0.02	−0.16^*^	0.72^**^	−0.40^**^	0.38^**^	0.02	0.42^**^	0.52^**^
20. Exhaustion T2	3.15	1.10	−0.12	0.32^**^	0.19^**^	−0.16^*^	−0.20^**^	−0.13	−0.02	0.30^**^	−0.41^**^	0.70^**^	−0.21^**^	0.37^**^	−0.24^**^	−0.30^**^
21. Social support T3	3.06	0.55	0.69^**^	−0.02	0.16^*^	0.16^*^	0.14	0.03	0.07	−0.16^*^	0.33^**^	−0.12	0.74^**^	−0.08	0.25^**^	0.37^**^
22. Psychological demands T3	2.84	0.50	0.03	0.67^**^	−0.06	0.11	0.12	0.18^**^	0.05	0.21^**^	0.06	0.28^**^	−0.07	0.74^**^	0.01	0.06
23. Mindfulness T3	2.68	0.45	0.20^**^	0.03	0.68^**^	0.37^**^	0.27^**^	0.26^**^	0.21^**^	−0.14*	0.31^**^	−0.21^**^	0.27^**^	−0.08	0.78^**^	0.39^**^
24. Incr. structural resources T3	3.88	0.57	0.24^**^	0.08	0.24^**^	0.54^**^	0.18^**^	0.23^**^	0.09	−0.08	0.34^**^	−0.07	0.27^**^	−0.03	0.36^**^	0.59^**^
25. Incr. social resources T3	2.67	0.82	0.17^*^	0.03	0.10	0.16^*^	0.69^**^	0.25^**^	0.11	0.09	0.30^**^	−0.21^**^	0.17^*^	0.05	0.21^**^	0.21^**^
26. Incr. challenging demands T3	3.03	0.77	0.08	0.15^*^	0.17^*^	0.31^**^	0.34^**^	0.63^**^	0.13	0.04	0.37^**^	−0.18^*^	0.07	0.07	0.35^**^	0.40^**^
27. Decr. hindering demands T3	3.03	0.79	0.19^**^	−0.01	0.08	0.12	0.15^*^	0.15^*^	0.48^**^	0.04	0.12	−0.13	0.15^*^	−0.05	0.22^**^	0.29^**^
28. Self-undermining T3	3.19	0.80	−0.23^**^	0.21^**^	−0.18*	−0.18^**^	−0.08	0.00	−0.09	0.66^**^	−0.29^**^	0.24^**^	−0.25^**^	0.23^**^	−0.27^**^	−0.28^**^
29. Engagement T3	4.36	1.11	0.31^**^	−0.02	0.21^**^	0.35^**^	0.34^**^	0.28^**^	−0.02	−0.04	0.73^**^	−0.34^**^	0.33^**^	−0.01	0.34^**^	0.44^**^
30. Exhaustion T3	3.29	1.14	−0.14^*^	0.32^**^	−0.15^*^	−0.12	−0.20^**^	−0.08	0.04	0.16^*^	−0.34^**^	0.56^**^	−0.21^**^	0.34^**^	−0.21^**^	−0.24^**^

Variable	15	16	17	18	19	20	21	22	23	24	25	26	27	28	29	30
15. Incr. social resources T2	(0.70)															
16. Incr. challenging demands T2	0.36^**^	(0.63)														
17. Decr. hindering demands T2	0.25^**^	0.36^**^	(0.67)													
18. Self-undermining T2	0.08	−0.08	−0.19^**^	(0.73)												
19. Engagement T2	0.31^**^	0.42^**^	0.11	−0.24^**^	(0.92)											
20. Exhaustion T2	−0.10	−0.16^*^	−0.05	0.38^**^	−0.47^**^	(0.81)										
21. Social support T3	0.18*	0.08	0.03	−0.16^*^	0.40^**^	−0.17*	(0.85)									
22. Psychological demands T3	0.07	0.14^*^	0.05	0.25^**^	0.07	0.37^**^	−0.05	(0.81)								
23. Mindfulness T3	0.19^**^	0.30^**^	0.17^*^	−0.10	0.38^**^	−0.28^**^	0.32^**^	−0.05	(0.87)							
24. Incr. structural resources T3	0.17^*^	0.34^**^	0.16^*^	−0.08	0.45^**^	−0.20^**^	0.36^**^	0.07	0.54^**^	(0.75)						
25. Incr. social resources T3	0.74^**^	0.30^**^	0.20^**^	0.09	0.37^**^	−0.17^*^	0.29^**^	0.06	0.28^**^	0.28^**^	(0.77)					
26. Incr. challenging demands T3	0.40^**^	0.74^**^	0.30^**^	−0.02	0.47^**^	−0.26^**^	0.20^**^	0.13	0.45^**^	0.49^**^	0.49^**^	(0.68)				
27. Decr. hindering demands T3	0.19^**^	0.21^**^	0.64^**^	−0.08	0.21^**^	−0.19^**^	0.28^**^	−0.02	0.33^**^	0.40^**^	0.31^**^	0.35^**^	(0.71)			
28. Self-undermining T3	−0.08	−0.14	−0.19^**^	0.66^**^	−0.29^**^	0.37^**^	−0.31^**^	0.28^**^	−0.24^**^	−0.27^**^	−0.07	−0.10^*^	−0.15^*^	(0.72)		
29. Engagement T3	0.34^**^	0.40^**^	0.06	−0.14*	0.80^**^	−0.41^**^	0.42^**^	0.00	0.41^**^	0.51^**^	0.46^**^	0.52^**^	0.27^**^	−0.25^**^	(0.93)	
30. Exhaustion T3	−0.18^*^	−0.15^*^	−0.06	0.29^**^	−0.37^**^	0.66^**^	−0.30^**^	0.42^**^	−0.27^**^	−0.27^**^	−0.26^**^	−0.22^**^	−0.26^**^	0.40^**^	−0.47^**^	(0.85)

Cronbach’s alphas are reported in parentheses. Incr., increasing; Decr., decreasing. ^*^p < 0.05, ^**^p < 0.01.

### 4.1 Test of hypotheses

#### 4.1.1 Study and personal resources and study demands

Social support from lecturers significantly decreased from T1 to T2, from T1 to T3, and from T2 to T3 in the WLC, but not in the IGs, which provides partial support for H1a, even though the time x group interaction effect was not significant.

For mindfulness, we found a statistically significant time × group interaction effect. The main time effects show that in both IGs, mindfulness was higher at T2 and T3 compared to T1, whereas in the WLC, we did not observe a significant change in mindfulness across time points. These results fully support H1b.

Psychological demands significantly increased in the WLC from T1 to T2 and from T1 to T3, but not in the IGs, which provides partial support for H1c, even though the time × group interaction effect was not significant.

#### 4.1.2 Engagement and exhaustion

For engagement, Levene’s test was significant (*p* < 0.01 for T1 and T2), so we performed a Welch-Test as a robust alternative. We found no statistically significant time x group interaction effect and no significant main time effect, so we reject H2a.

For exhaustion, we found a statistically significant time x group interaction effect. The main time effects show that in the WLC, exhaustion was higher at T2 and T3 compared to T1, whereas in both IGs, we did not observe a significant change in exhaustion across time points. Therefore, the significant intervention effect did not result from the hypothesized reduction in exhaustion within the IGs, but rather from the increase observed in the WLC, which was counteracted by the intervention. Thus, we partially confirm H2b.

#### 4.1.3 Study crafting and self-undermining

For increasing structural resources, we found a statistically significant time x group interaction effect. The main time effects show that in the WLC, increasing structural resources was lower at T3 compared to T1, whereas in both IGs, we did not observe a significant change in this study crafting strategy across time points. For increasing social resources, we found no statistically significant time x group interaction effect and no significant main time effect. For increasing challenging demands, we found a statistically significant time x group interaction effect. The main time effects show that in IG2, increasing challenging demands was higher at T3 compared to T1 and T2, whereas in IG1 and the WLC, we did not observe a significant change in this study crafting strategy across time points. For decreasing hindering demands, we found no statistically significant time x group interaction effect, but a significant main time effect. In IG1, decreasing hindering demands increased from T1 to T2 and from T1 to T3 and in the WLC, decreasing hindering demands increased from T1 to T3, whereas in IG2, we did not observe a significant change in this study crafting strategy. These results partially support H3a.

For self-undermining, we found a statistically significant time × group interaction effect. The main time effects show that in IG2, self-undermining decreased from T2 to T3 and in the WLC, self-undermining increased from T1 to T3 and from T2 to T3. In IG1, we did not observe a significant change in self-undermining across time points. Thus, we partially confirm H3b.

Tables 3 and 4 show the means and standard deviations of the study variables for the three groups and the results of the RM ANOVA (interaction effects and main time effects). Supplementary Table S1 shows the time effects for each of the two measurement time points for the three groups. Supplementary Figure S1 shows the results for variables with significant interaction effects and/or main time effects graphically.

**Table 3:** Means and standard deviations for all study variables at the three measurement time points for the three groups.

Variable	IG1			IG2			WLC		
	T1	T2	T3	T1	T2	T3	T1	T2	T3
	*M* (*SD*)	*M* (*SD*)	*M* (*SD*)	*M* (*SD*)	*M* (*SD*)	*M* (*SD*)	*M* (*SD*)	*M* (*SD*)	*M* (*SD*)
Social support	3.18 (0.50)	3.11 (0.50)	3.16 (0.57)	3.17 (0.46)	3.08 (0.50)	3.09 (0.53)	3.15 (0.48)	3.05 (0.49)	2.95 (0.52)
Mindfulness	2.60 (0.48)	2.73 (0.43)	2.75 (0.43)	2.61 (0.38)	2.74 (0.42)	2.73 (0.41)	2.61 (0.44)	2.58 (0.44)	2.57 (0.49)
Psychological demands	2.73 (0.52)	2.77 (0.45)	2.80 (0.49)	2.75 (0.50)	2.78 (0.42)	2.83 (0.46)	2.74 (0.53)	2.87 (0.52)	2.88 (0.55)
Engagement	4.47 (1.06)	4.41 (1.16)	4.49 (1.22)	4.35 (0.79)	4.46 (0.80)	4.39 (1.02)	4.30 (1.13)	4.15 (1.18)	4.21 (1.09)
Exhaustion	3.03 (1.03)	2.98 (1.13)	3.02 (1.22)	3.25 (1.02)	3.13 (0.91)	3.26 (0.98)	3.07 (1.11)	3.33 (1.23)	3.56 (1.17)
Increasing structural resources	3.90 (0.47)	3.92 (0.46)	3.95 (0.46)	3.93 (0.51)	3.89 (0.51)	3.96 (0.55)	3.93 (0.54)	3.81 (0.55)	3.74 (0.65)
Increasing social resources	2.67 (0.81)	2.71 (0.76)	2.83 (0.87)	2.53 (0.65)	2.60 (0.77)	2.63 (0.84)	2.58 (0.70)	2.52 (0.71)	2.58 (0.73)
Increasing challenging demands	3.00 (0.74)	2.98 (0.78)	3.11 (0.78)	2.83 (0.77)	2.95 (0.78)	3.11 (0.71)	2.94 (0.79)	2.89 (0.71)	2.89 (0.80)
Decreasing hindering demands	2.75 (0.94)	3.07 (0.82)	3.12 (0.74)	2.79 (0.72)	2.94 (0.74)	3.01 (0.85)	2.72 (0.82)	2.86 (0.76)	2.96 (0.78)
Self-undermining	3.07 (0.72)	3.09 (0.73)	3.14 (0.76)	2.98 (0.84)	3.15 (0.88)	2.95 (0.69)	3.25 (0.86)	3.32 (0.79)	3.47 (0.85)

**Table 4:** Results of the RM ANOVA.

Variable	RM-ANOVA			
	Time × group interaction effect		Main time effect	
	*F*-value	*η*_p_^2^	*F*-value	*η*_p_^2^
Social support	*F*(4, 404) = 2.24, *p* = 0.06		*F*(2, 404) = 8.49, *p* < 0.001	0.04
Mindfulness	*F*(3.95, 398.52) = 4.70, *p* < 0.01	0.04		
Psychological demands	*F*(3.94, 397.95) = 0.75, *p* = 0.56		*F*(1.97, 397.95) = 6.80, *p* < 0.01	0.03
Engagement	Welch’s *F*(4, 316.5) = 0.61, *p* = 0.66		Welch’s *F*(2, 404.8) = 0.24, *p* = 0.79	
Exhaustion	*F*(3.88, 392.20) = 3.63, *p* < 0.01	0.04		
Increasing structural resources	*F*(4, 404) = 2.58, *p* < 0.05	0.03		
Increasing social resources	*F*(4, 404) = 0.88, *p* = 0.48		*F*(2, 404) = 2.32, *p* = 0.10	
Increasing challenging demands	*F*(3.91, 394.78) = 2.69, *p* < 0.05	0.03		
Decreasing hindering demands	*F*(3.85, 389.18) = 0.67, *p* = 0.61		*F*(1.93, 389.18) = 14.59, *p* < 0.001	0.07
Self-undermining	*F*(4, 404) = 3.02, *p* < 0.05	0.03		

#### 4.1.4 Mediation

We found no significant indirect effects through the study resource social support from lecturers and the study demand psychological demands. Thus, we reject H5. However, the intervention significantly predicted the personal resource mindfulness (T2) as a mediator, *b* = 0.17, *p* < 0.001. Mindfulness, in turn, significantly predicted engagement (T3), *b* = 0.53, *p* < 0.01, increasing structural resources (T3), *b* = 0.32, *p* < 0.01, increasing challenging demands (T3), *b* = 0.52, *p* < 0.001, and self-undermining (T3), *b* = −0.41, *p* < 0.01, but not exhaustion, *b* = −0.34, *p* = 0.12. Thus, we partially confirm H4a, H4c, and H4d and reject H4b. The significant indirect effects are shown in Table 5.

**Table 5:** Overview of the indirect effects.

Variable	Engagement		Increasing structural resources		Increasing challenging demands		Self-undermining	
	*B(SE)*	95%CI	*B(SE)*	95%CI	*B(SE)*	95%CI	*B(SE)*	95%CI
Mindfulness	0.09(0.04)	0.019, 0.178	0.05(0.02)	0.014, 0.106	0.09(0.03)	0.031, 0.159	−0.07(0.03)	−0.137, −0.016

## 5 Discussion

Given the increase in burnout and the temporary decrease in engagement during the COVID-19 pandemic (e.g., Herbst et al., 2016; Salmela-Aro et al., 2022), there is an urgent need for evidence-based support programs for students. Our study therefore tested a novel intervention approach by evaluating two online interventions based on the SD-R framework, aimed at balancing resources and demands. Consistent with our hypotheses, the interventions positively influenced the personal resource mindfulness, two study crafting strategies, self-undermining, and exhaustion. The hypothesized mediation effects through the personal resource mindfulness were partially confirmed.

While we observed no significant interaction effects for the study resource and study demand, there were significant time effects within the WLC. Social support from lecturers decreased over time, while psychological demands increased. Since lectures switched from face-to-face (T1) to online (T2 and T3) during the course of our study, these results align with research suggesting that social support decreased and workload increased during the COVID-19 pandemic, especially with the shift to online lectures (Hoss et al., 2021). The results also align with literature indicating workload intensification throughout the semester (Pitt et al., 2018). Contrary to our hypotheses, social support did not increase and psychological demands did not decrease in the IGs, but remained stable. This at least suggests that our interventions may help counteract the loss of resources and the increase in demands typically associated with online lectures and semester progression. A job crafting intervention study also found no effect on job resources and demands after the intervention, but increased job resources at the 1-year follow-up (van Wingerden et al., 2017b). Accordingly, changes in the study environment may take time to become measurable.

The personal resource mindfulness significantly increased in both IGs after the intervention and compared to the WLC, with effects persisting at follow-up. This supports the notion that actively engaging with one’s own demands and resources can sustainably enhance mindfulness. Given its positive correlation with life satisfaction, optimism, and self-esteem and its negative correlation with negative affect (Brown and Ryan, 2003), this is a promising result. Consistent with our findings, job crafting interventions also increased personal resources such as self-efficacy (van Wingerden et al., 2017b) or psychological capital (van Wingerden et al., 2016).

Unexpectedly, we did not observe an intervention effect on engagement, which is in contrast to an earlier study on a study crafting intervention (Körner et al., 2022). However, job crafting interventions also appear to have heterogeneous effects on work engagement (Devotto and Wechsler, 2019). A meta-analysis concludes that the resource gain appears to be a key condition for increasing engagement through interventions (Devotto and Wechsler, 2019). The COVID-19 pandemic and the change to online lectures after the intervention eliminated some resources (Lederer et al., 2021). Salta et al. (2022) also noted lower engagement in online lectures versus face-to-face lectures. Thus, our result may be explained by a lack of sufficient resource building of the participants as well as the switch to online lectures.

A significant intervention effect was observed on exhaustion. Exhaustion significantly increased in the WLC, which is consistent with studies confirming that stressors accumulate and negative stress-related outcomes occur particularly at the end of the semester (Pitt et al., 2018). In addition, Salmela-Aro et al. (2022) confirmed that exhaustion increased steadily over the course of the COVID-19 pandemic. In contrast, exhaustion remained stable in both IGs, suggesting that our interventions might help counteract increasing exhaustion as the semester progresses. A study crafting intervention study found a significant decrease of exhaustion at the 5-month follow-up in the IG (Körner et al., 2022). This suggests that it may take longer for exhaustion to decrease measurably as a result of an intervention.

Regarding study crafting, significant intervention effects were found for increasing structural resources and increasing challenging demands. In the WLC, increasing structural resources significantly decreased, while there were no significant changes in both IGs. As stressors accumulate over the course of the semester (Pitt et al., 2018), students may feel too stressed to increase structural resources. Our intervention may have helped encourage students to engage in this behavior even during stressful times. van Wingerden et al. (2017a) posited that increasing structural resources requires ample opportunities and time for successfully implementation. The COVID-19 pandemic has eliminated some structural resources that can contribute to personal development such as a semester abroad (Lederer et al., 2021). This may also explain why this study crafting strategy did not increase in the IGs. Increasing challenging demands significantly increased in IG2, possibly due to the focus on resources in this group, which may have strengthened participants to seek new challenges.

No intervention effect was found on increasing social resources, aligning with a job crafting intervention review (Devotto and Wechsler, 2019), which also reported no effect on this strategy. Students reported a decrease in interaction, communication, and support due to the COVID-19 pandemic (Hoss et al., 2021). This might have limited students’ opportunities to increase their social resources during online lectures at T2 and T3.

Decreasing hindering demands significantly increased in IG1, consistent with the results of a study crafting and some job crafting interventions (van Wingerden et al., 2017a; Körner et al., 2022). However, as this strategy also increased in the WLC, it seems partly intuitive, particularly during stressful periods like the shift to online lectures and exam preparation. Studies from the work context confirm that decreasing hindering demands is an effective strategy especially during stressful times (Demerouti et al., 2017). Interestingly, in IG2, this behavior remained stable, possibly due to an increased capacity to cope with stressors due to their focus on resources.

For self-undermining, an increase was observed in the WLC, while it decreased in IG2 from T2 to T3 and remained stable in IG1. As this behavior has not yet been investigated in intervention contexts, our results provide novel insights, suggesting that SD-R-based interventions can mitigate dysfunctional behaviors that typically increase over the course of a semester.

Our mediation analyses indicated that mindfulness mediated the effect of the interventions on several SD-R outcomes. This is in line with the assumptions of a gain cycle between resources, engagement, and job crafting, as well as the buffer hypothesis postulating that resources can also impact variables of the health-impairment path (Bakker and Demerouti, 2014, 2017). Our study confirms that mindfulness can help promoting engagement and study crafting, while countering self-undermining. This further confirms the suitability of integrating it within the SD-R framework, given its influence on both the health-impairment path and the motivational path.

### 5.1 Theoretical contributions

Our study contributes to the literature in three significant ways: First, it extends the literature on the SD-R framework by demonstrating that SD-R-based interventions can actively influence the postulated paths. Since we confirmed intervention effects on mindfulness, study crafting, and self-undermining in addition to exhaustion, our study also contributes to the validation of an extended SD-R framework. Additionally, our mediation analyses underscore the importance of personal resources within the SD-R framework, highlighting their role in influencing both behavioral (i.e., study crafting, self-undermining) and well-being outcomes (i.e., engagement).

Second, our study contributes to the literature on interventions in higher education settings. While existing reviews indicate heterogeneous effects of online stress management or mindfulness interventions (e.g., Harrer et al., 2019; Dawson et al., 2020), our findings indicate that the SD-R framework represents a novel effective intervention approach. Thereby, our study also addresses the critical need for further evidence-based support programs for students (Mülder et al., 2022).

Third, our research adds to the understanding of proactive and dysfunctional student behavior. A study crafting intervention only increased the study crafting strategy of decreasing hindering demands (Körner et al., 2022), while our intervention had an impact on a broader range of study crafting strategies (i.e., increasing structural resources and increasing challenging demands). This suggests that the intervention focus on balancing resources and demands can achieve different effects than a study crafting intervention. Moreover, our study is pioneering in demonstrating that interventions can positively affect self-undermining, which, to our knowledge, has not been previously investigated in an intervention in either the academic context or work context.

### 5.2 Limitations and suggestions for further research

Although our study was carefully planned and conducted, it has some limitations that should be considered in future research: First, we focused on a limited set of study demands, study resources, and personal resources, with some showing no significant intervention effects. Future studies could explore the impact of SD-R-based interventions on a broader range of study resources (e.g., qualification potential) and study demands (e.g., incompatibility of study and private life) (Gusy et al., 2016). Similarly, the impact of these interventions on other outcome variables that have already been examined in cross-sectional studies in the context of the SD-R framework such as life satisfaction or performance could be examined (Schaufeli et al., 2002; Lesener et al., 2020).

Second, currently there are no validated scales for assessing study crafting and self-undermining among students. Therefore, scales developed and validated for the work context were adapted to the higher education context. Although most scales exhibited satisfactory reliability, the results concerning study crafting and self-undermining should be interpreted with caution. Therefore, future research should prioritize the development and validation of such measurement instruments.

Third, the changing study conditions during the course of our study (online vs. face-to-face lectures, exam phase at follow-up) may also have influenced our results, as discussed earlier. Future research should consider varying time points and examine the longer-term impact of SD-R-based interventions (i.e., one semester later) as some effects may only emerge over time, as evidenced in a job crafting intervention study in the work context (van Wingerden et al., 2017b).

Fourth, our study’s generalizability is limited by the specific demographic composition of the sample. The sample consisted predominantly of women and bachelor’s students at a single university of applied sciences. At the same time, Herbst et al. (2016) emphasize that women show higher stress levels compared to men, students at universities of applied sciences compared to students at universities, and bachelor’s students compared to master’s students. Thus, our intervention targeted a particularly vulnerable population, which has a high practical value for this target group, but further limits its transferability of the results. Future studies could address these points and also investigate whether sociodemographic variables or personality traits (e.g., regulatory focus) influence how students respond to the resources-intervention compared to the demands-intervention.

Last, self-report data, while appropriate for capturing subjective well-being and personal perceptions regarding the own study environment, is susceptible to common method bias (Podsakoff et al., 2003). Future studies might incorporate peer or lecturer ratings for a more comprehensive assessment of certain variables.

### 5.3 Practical implications and conclusion

Our study demonstrated that SD-R-based interventions are effective in enhancing mindfulness and study crafting behavior, and mitigating self-undermining and exhaustion among students. Notably, our study underscores that brief interventions, requiring only 3–4 h over 2 weeks, can yield positive outcomes, which also meets students’ preference for compact stress management solutions and online interventions (Seidl et al., 2018; Harrer et al., 2019). Therefore, a key practical implication of our study is the great potential of our interventions for higher education institutions as a cost-effective, and time- and resource-efficient way to support students. A notable observation is that the intervention group focusing on resources (IG2) exhibited more positive intervention effects. This suggests that interventions emphasizing resource enhancement are particularly impactful.

However, beyond implementing targeted interventions, it is crucial for higher education institutions to create a study environment that facilitates the adjustment of resources and demands. This involves creating opportunities for personal growth and autonomy, such as diverse extracurricular activities or flexible course choices. In addition, lecturers should pay attention to fostering social resources such as communication, support, feedback, and interaction. To counteract accumulation of demands and associated stress, higher education institutions should also ensure a distribution of academic demands across the semester, for example, by staggering exam schedules and assignment deadlines.

In conclusion, it should be noted that demands and resources can fluctuate greatly over the course of a semester, and study conditions can change rapidly due to external circumstances such as the COVID-19 pandemic (Körner et al., 2021). The SD-R framework provides an adaptable and responsive basis for interventions that can help customize demands and resources to respond flexibly to changing circumstances. While our intervention warrants further refinement and testing, we conclude that SD-R-based interventions offer a promising measure for improving student well-being by creating an optimal balance of demands and resources.

## Data availability statement

The raw data supporting the conclusions of this article will be made available by the authors, without undue reservation.

## Ethics statement

Ethical approval was not required for the studies involving humans in accordance with the local legislation and institutional requirements. The participants provided their written informed consent to participate in this study.

## Author contributions

LK: Conceptualization, Formal analysis, Investigation, Methodology, Project administration, Writing – original draft, Data curation, Visualization. TK: Conceptualization, Project administration, Resources, Writing – review & editing, Formal analysis, Methodology. KR: Writing – review & editing, Conceptualization, Methodology. TR: Supervision, Writing – review & editing.

## Footnotes

^1^The ZPP is a cooperative association of statutory health insurance funds in Germany, which reviews and certifies preventive health programs based on the “Leitfaden Prävention” [Prevention Guideline] in accordance with uniform qualitative standards.
