# Supplementary material for: Evaluation of two study demands-resources-based interventions: a randomized controlled trial
Source: Front Psychol. 2024 Jun 10;15:1368267. doi: 10.3389/fpsyg.2024.1368267 (PMC11194431; doi:10.3389/fpsyg.2024.1368267)
Supplement: Supplementary file 1 [file Table_1.pdf]

Supplementary Table S1. Significant time effects between each of the two measurement time points for the three groups.

Variable	Group	T1-T2		T1-T3		T2-T3	
		F-value	η_p^2	*F*-value	η_p^2	*F*-value	η_p^2
Social support	WLC	$F(1, 70) = 5.66, p < 0.05$	0.08	$F(1, 70) = 21.06, p < 0.001$	0.23	$F(1, 70) = 5.00, p < 0.05$	0.07
Mindfulness	IG1	$F(1, 63) = 10.10, p < 0.01$	0.14	$F(1, 63) = 15.99, p < 0.001$	0.20		
	IG2	$F(1, 69) = 11.89, p < 0.001$	0.15	$F(1, 69) = 9.81, p < 0.01$	0.12		
Psychological demands	WLC	$F(1, 70) = 8.10, p < 0.01$	0.10	$F(1, 70) = 7.09, p < 0.05$	0.09		
Exhaustion	WLC	$F(1, 70) = 5.38, p < 0.05$	0.07	$F(1, 70) = 12.36, p < 0.001$	0.15		
Incr. structural resources	WLC			$F(1, 70) = 8.78, p < 0.01$	0.11		
Incr. challenging demands	IG2			$F(1, 69) = 12.77, p < 0.001$	0.16	$F(1, 69) = 6.73, p < 0.05$	0.09
Decr. hindering demands	IG1	$F(1, 63) = 11.84, p < 0.01$	0.16	$F(1, 63) = 14.18, p < 0.001$	0.18		
	WLC			$F(1, 70) = 5.53, p < 0.05$	0.07		
Self-undermining	IG2					$F(1, 66) = 4.83, p < 0.05$	0.07
	WLC			$F(1, 70) = 7.27, p < 0.01$	0.09	$F(1, 70) = 4.94, p < 0.05$	0.07

Empty cells indicate non-significant effects. Incr. = Increasing, Decr. = Decreasing.
