# Supplementary material for: Evaluation of two study demands-resources-based interventions: a randomized controlled trial
Source: Front Psychol. 2024 Jun 10;15:1368267. doi: 10.3389/fpsyg.2024.1368267 (PMC11194431; doi:10.3389/fpsyg.2024.1368267)
Supplement: Supplementary file 2 [file Image_1.pdf]

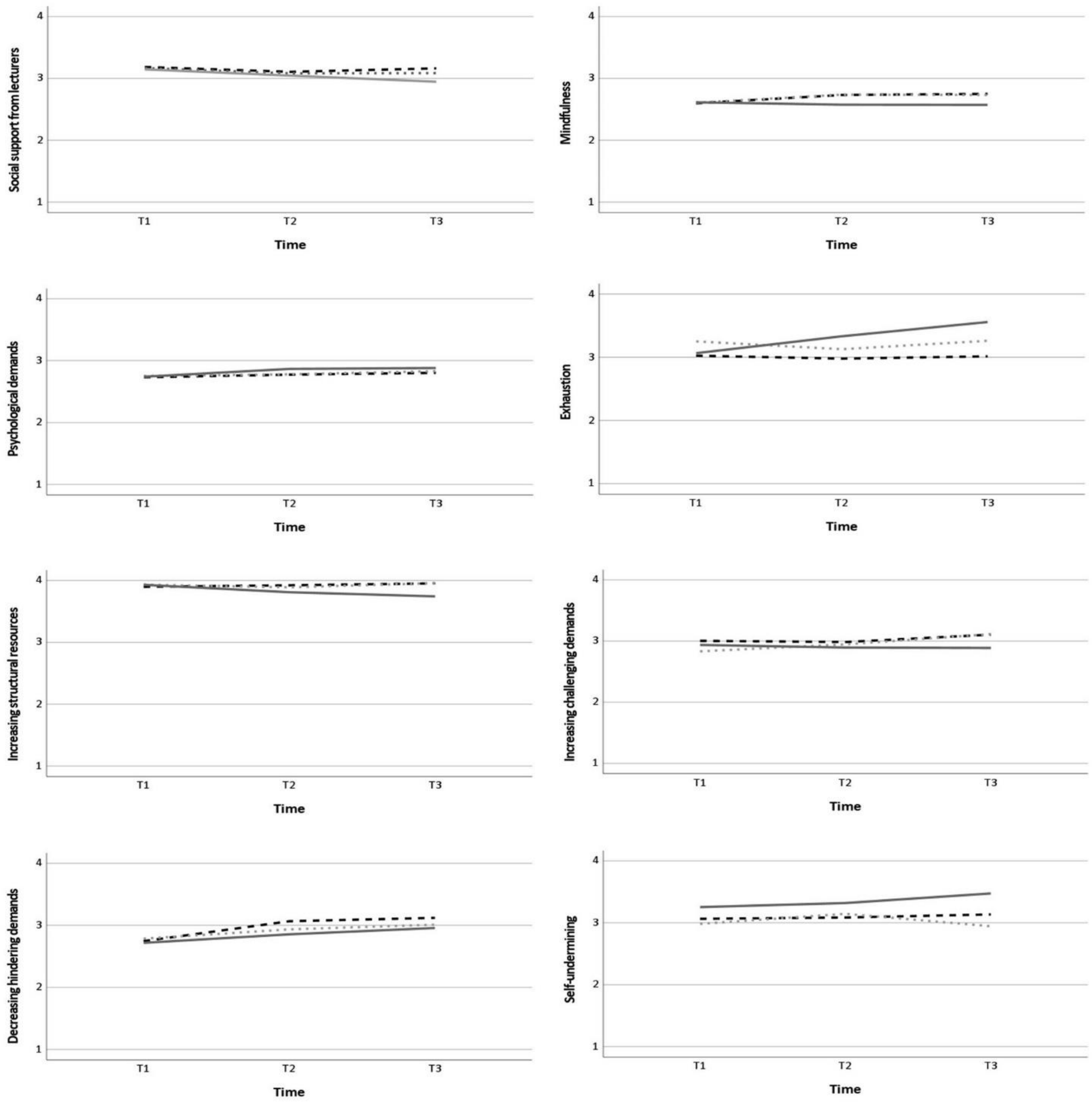

Supplementary Figure S1.
Results of the RM ANOVA for the study variables with significant interaction effects or significant main time effects. IG1 - - - IG2 ········ WLC ——
